# Beneficial versus Detrimental Effects of Complement–Microglial Interactions in Alzheimer’s Disease

**DOI:** 10.3390/brainsci14050434

**Published:** 2024-04-26

**Authors:** Gunel Ayyubova, Nadeem Fazal

**Affiliations:** 1 Department of Cytology, Embryology and Histology, Azerbaijan Medical University, Baku 370022, Azerbaijan; gunel.ayubova@gmail.com; 2 College of Health Sciences and Pharmacy, Chicago State University, Chicago, IL 60628, USA

**Keywords:** complement proteins, cognitive decline, synapse loss, microglia, neurodegeneration

## Abstract

Research indicates that brain-region-specific synapse loss and dysfunction are early hallmarks and stronger neurobiological correlates of cognitive decline in Alzheimer’s disease (AD) than amyloid plaque and neurofibrillary tangle counts or neuronal loss. Even though the precise mechanisms underlying increased synaptic pruning in AD are still unknown, it has been confirmed that dysregulation of the balance between complement activation and inhibition is a crucial driver of its pathology. The complement includes three distinct activation mechanisms, with the activation products C3a and C5a, potent inflammatory effectors, and a membrane attack complex (MAC) leading to cell lysis. Besides pro-inflammatory cytokines, the dysregulated complement proteins released by activated microglia bind to amyloid β at the synaptic regions and cause the microglia to engulf the synapses. Additionally, research indicating that microglia-removed synapses are not always degenerating and that suppression of synaptic engulfment can repair cognitive deficits points to an essential opportunity for intervention that can prevent the loss of intact synapses. In this study, we focus on the latest research on the role and mechanisms of complement-mediated microglial synaptic pruning at different stages of AD to find the right targets that could interfere with complement dysregulation and be relevant for therapeutic intervention at the early stages of the disease.

## 1. Introduction

Alzheimer’s disease (AD) is a neurodegenerative disease that causes large numbers of amyloid plaques and neurofibrillary tangles to build up in the brain. Its main pathological features include the abnormal deposition of extracellular amyloid β plaques and the intracellular neurofibrillary tangles of tau proteins. It is one of the leading causes of illness and death around the world. Given the absence of effective therapeutic methods for and the mostly unexplained etiology of AD, scientists have been pushed to look deeper into molecular mechanisms and pathways to comprehend the disease’s pathophysiology. It has been established that innate immune responses play a significant role in AD development and progression. Genome-wide association studies (GWASs) have revealed many AD risk genes that converge in microglial cell phagocytic pathways [1,2]. Among the risk variations are single-nucleotide polymorphisms (SNPs) in complement receptor 1 (CR1), clusterin (CLU) [2], and C1S [3].

It is commonly known that the complement system is a key component of the innate immune response and a strong inducer of neuroinflammation in AD. Increasing evidence shows that the amyloid β protein (Aβ) stimulates complement activity [4,5], which causes localized chronic inflammation and activation of the glial cells in the vicinity [6]. By co-localizing with amyloid β in the capillaries of the brain, complement proteins can cause cerebral amyloid angiopathy [7]. On the contrary, the levels of complement regulators significantly decrease in AD brains, which emphasizes potentially significant disturbances in the regulation of inflammatory reactions. The continuous production of inflammatory mediators and complement proteins by the microglia exaggerates amyloid pathology. It leads to a self-supported neuroinflammation loop between the overactivated microglia, the complement system, and Aβ plaques, worsening AD’s pathology. Microglial autophagy and the inflammatory response are critical for protection against external insults. Deficiency in microglial autophagy leads to the development of many neurodegenerative disorders. Complement proteins function as “eat me” signals, which can mark apoptotic cells for removal by the microglia, as they are the only CNS cell type that expresses CR3 receptors [8]. 

Complement-dependent glial-mediated synaptic pruning has been shown to be among the primary contributors to the loss of synapses at the early stages of AD [9]. Glial cells isolated from AD brains had higher concentrations of synaptic proteins than microglia and astrocytes from non-AD individuals [10,11]. Additionally, aberrant tau oligomers in the synapses, as well as the presence of amyloid pathology, enhance glial-mediated synapse removal in the brains of AD patients [10,11]. In culture, synapses obtained from AD patients were more easily phagocytosed by primary mouse and human microglia and astrocytes than synapses derived from control brains. Research has also shown that inhibiting the opsonophagocytic mechanism can regulate glial synapse ingestion [11]. Additionally, recent research has shown that the excessive synaptic loss associated with several neurodegenerative diseases can be reduced by the deletion of not only C1q but also the C3 and C4 complement proteins [12,13]. These findings imply that the opsonophagocytic mechanism, which involves the activation of complement components, is crucial to the internalization of synapses by the astrocytes and microglia in human AD brains and mouse models.

However, these indications of the role of complement activation in the acceleration of AD progression and evidence that suppressing the complement system is not without risk raise the idea that targeting the complement, especially in the brain, requires closer attention. Therefore, important issues on complement-mediated synapse loss in AD still need to be resolved. Moreover, it is unclear what physiological processes in AD make the synapses more susceptible to being “tagged”. In this article, we explain how microglia–complement interactions affect brain functions while also playing a role in synaptic loss. Also, we present an overview of the recent findings on complement-mediated synaptic decline in AD and provide clarification regarding its underlying mechanism.

## 2. Complement Proteins

The complement is a crucial element of the innate immune system that protects the body from pathogens and injured cells [14,15]. It promotes inflammatory reactions through the generation of anaphylatoxins [16]. The complement includes an enzymatic cascade with approximately 50 proteins and membrane receptors [6,16]. The classical, lectin, and alternative pathways are well-known mechanisms for complement activation [17].

The classical pathway is activated when C1 (C1qrs) interacts with immune complexes and microbes, as well as specific proteins (such as Aβ or α-synuclein protein). All three pathways join to form a C3 convertase, which then cleaves C3 to release C3a and C3b. The latter then cleaves C5 into C5a and C5b. As anaphylatoxins that provide immune cell recruitment and inflammation, C3a and C5a [17] act via binding to their respective receptors. The C3a receptor (C3aR) and the C5a receptor (C5aR1) are present on the membranes of neutrophils and macrophages. The second C5a-like receptors for binding with C5a are C5L2 and C5aR2. The binding of C3b to C3R on the surface of phagocytes leads to an increase in their phagocytic activity [18]. In contrast, the activation of C5aR1 initiates pro-inflammatory responses via the stimulation of cytokine or chemokine production and the activation of phagocytes. Nevertheless, C3aR signaling is mainly immunomodulatory, and depending on the context, may exert pro- or anti-inflammatory effects [19,20]. Ricklin et al. [17] found that C5b encourages the formation of the membrane attack complex (MAC), which breaks down the integrity of the cellular plasma membrane, followed by cell lysis and death.

Different regulators or controllers of complement activation (RCAs), such as factor H (FH), factor I, C1-inhibitor, clusterin, etc., function to limit the complement activation both in time and place and prevent autologous tissue damage. When an immune response takes place against a pathogen, the complement system is activated and triggers an inflammatory response that helps the immune cells combat infection by increasing or “complementing” the capacity of antibodies and phagocytic cells to eliminate microbes and injured cells.

Despite the well-known functions of the complement system in the peripheral organs, its roles in the central nervous system (CNS) are still obscure and under investigation. In the CNS, complement proteins, as well as their regulatory proteins and receptors, are secreted primarily by glial cells and neurons [21]. The CNS uses the complement for several crucial processes, including synaptic plasticity throughout life, synapse elimination, neurogenesis, apoptosis, and neuronal plasticity. In the process of synaptic pruning during development, complement proteins contribute to the elimination of inactive synapses to allow the strengthening and maturing of more healthy connections [22].

**Figure 1:**
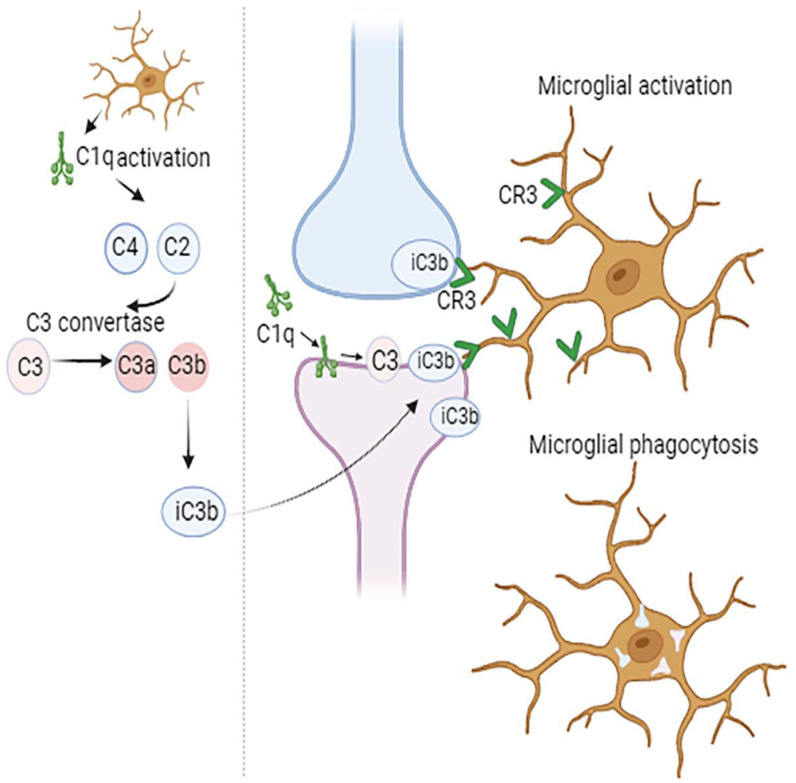
Complement-mediated synapse elimination. The initial step in synapse pruning involves the production of complement component C1q by glial cells. This is followed by cleavage of C4 and C2, activation of C3 convertase, and production of C3a and C3b. Then, the opsonin iC3b, which is formed from C3b, is recognized by the CR3 receptor on microglia. Subsequent microglial activation leads to synapse phagocytosis.

The components of the classical complement cascade, representing a group of secreted “eat me” signals, mediate the recognition and phagocytosis of weak synapses by the microglia (Figure 1). This happens when synapses are tagged by C1q, opsonized by C3b, and then taken up by the microglia through CR3 activation [22,23]. The quantity of phagocytic microglia and the degree of early synapse loss are greatly decreased when C1q, C3, or the complement receptor CR3 in the microglia is suppressed [12,22]. Although CNS complement is mostly produced by the microglia and astrocytes, significant levels of C1q and CR3 have been demonstrated to be secreted by the microglia. In microglial-specific conditional C1q knockout mice, even though the blood C1q levels remained stable, C1q was not detected in the brain tissue, indicating that microglia are the primary source of C1q in the CNS [24].

Disturbances in the regulation of the complement system in the CNS contribute to various neurological disorders, including neurodevelopmental disorders such as autism and schizophrenia [23,25,26], neurodegenerative diseases, diseases associated with disturbances in blood–brain barrier (BBB) functions, etc. Additionally, recent research has demonstrated the critical role of the complement cascade in the development of neurological complications due to SARS-CoV-2 infection [27,28].

## 3. The Microglia in Health and Disease

Microglia are macrophage-like cells providing immunological surveillance of the central nervous system (CNS). They are derived in the yolk sac and in mice colonize the neuroepithelium at embryonic day 10.5 [29,30]. Reaching the brain, they propagate, ramify, and scatter all over the CNS [29]. During a lifetime, microglial homeostasis maintains the balance between their proliferation and apoptosis [31]. The proliferation of the microglial cells is regulated by IL-34, IFN regulatory factor 8, the transcription factor PU.1, and macrophage colony-stimulating factor 1 receptor [31].

Microglial depletion studies have shown that the source of replenishment for a depleted microglial population is exclusively brain-resident cells and takes place via an IL-1α-dependent pathway [32]. However, research has confirmed that in diseased conditions associated with blood–brain barrier (BBB) breakdowns, such as brain ischemia, autoimmune encephalomyelitis, and whole-body radiotherapy 2 [32], the peripheral monocytes can penetrate the brain parenchyma and populate the microglial niche. These findings suggest that circulating monocytes may contribute to the pathophysiology of AD, where BBB breakdown is apparent [33].

As classic tissue-resident macrophages, the microglia play vital roles in CNS tissue maintenance, homeostasis, and health [34]. They dynamically survey the environment and provide brain protection, response to tissue injury, and repair by actively removing phagocytic cellular debris [1]. To morpho-functionally adapt to their environment, microglia use a whole arsenal of resources, including plasticity of the elements of their cytoskeleton, a system of intracellular proteins, and microglial receptors that recognize pathogen-associated molecular patterns and damage-associated molecular patterns [35]. Additionally, they can phagocytose and produce a variety of substances that are involved in tissue maintenance and immunological defense, including chemokines, cytokines, trophic factors, and nitric oxide.

Recent studies on developing and adult brains have shown that microglia are not just immune cells; they are involved in adult neurogenesis [36,37], the formation of the brain architecture and wiring neural circuits, and vasculature development [38]. Importantly, there is strong evidence supporting the role of the microglia in the regulation of synaptic plasticity, a crucial cellular mechanism involved in learning and memory [39]. These cells engulf the synapses and reshape synaptic connections during normal brain development and its decline. This process varies by developmental stage and brain region, as well as stage of disease [40].

The homeostatic state of the microglia in a healthy brain is supported by neuronal–microglial crosstalk, which includes the signaling pathways involving CD200–CD200R and CX3CL1–CX3CR1 [41]. However, in the brains of patients with AD, the expression of CD200, as well as the receptors CD200R and CX3CR1, is diminished, leading to a reduced physiological regulation of microglial behavior [42]. Hence, in response to persistent or chronic stimulation, the microglia adopt a significantly different signature from their homeostatic signature and undergo changes in their morphology, proteomic markers, and behavior. This significantly depends on the region affected, the stage of disease, and the severity of the pathogenic environment in the brain tissue.

In the context of AD, microglial priming is increasingly proposed as a prerequisite process when chronic low-level stimuli (such as systemic inflammation and aging) cause naive microglia to assume an altered state, resulting in an enhanced or inappropriate inflammatory reaction in response to repeated pathological stimulation [43]. Alterations of the microglial phenotype in AD lead to the activation of inflammasome signaling and the release of excessive inflammatory cytokines, which, in turn, promotes neurotoxicity and excessive microglial synapse elimination [12,44,45]. The findings have confirmed that neuroinflammation is an ongoing process that does not resolve on its own and is regarded as a critical driver of AD [46]. Moreover, a growing body of evidence suggests that caspase-1 activation and pro-inflammatory cytokine (such as IL-1β) production occur before AD pathology, showing that activation of the microglial NLRP3 inflammasome represents an early pathogenic event in AD [47].

On the other hand, individuals with disrupted microglial functioning (which could be acquired or correspond to hereditary susceptibility) experience cognitive loss at an earlier clinical stage of AD [48] than those with intact microglial function. Thus, microglial behavior partially determines the vulnerability of people to AD concerning more severe clinical manifestations at a particular stage of the disease. Additionally, in late-stage AD, ineffective Aβ clearance and tau fibrillation negatively impact the microglial defense activities and induce persistent harmful microglial activation, contributing to neurodegeneration [49].

## 4. The Activation of Complement Proteins in AD

Although the mechanisms involved in the contribution of the complement cascade as well as complement-related genes to AD pathogenesis are under investigation, findings on human brain tissue and animal models demonstrate that complement regulatory dysfunction plays a significant role in the development of AD [45]. Post-mortem studies of AD brains have demonstrated a dramatic increase in the activation of all complement components [50,51]. Indeed, higher levels of C1q, C3, and C4 have been found in different brain regions, including the temporal cortex, especially near Aβ plaques and tau aggregates [52,53]. Furthermore, distinct disease stages can be distinguished by elevated quantities of complement proteins and activating components in the bloodstream and cerebrospinal fluid (CSF). In this regard, products of complement and immune system dysregulation might serve as biomarkers for an early diagnosis of AD and as predictors of disease progression [54,55].

Triggers of pathological C1q upregulation in the surrounding microglia and complement cascade activation are oligomeric/fibrillar Aβ and hyperphosphorylated tau [52,54,56]. Additionally, a variety of other complementary components have been linked to AD. Among these are complement protein 3a (C3a) and its receptor C3aR, complement protein 5a (C5a) and its receptor C5aR1, the complex C5b-C9, complement component 9, factor B, and factor D [57,58,59,60]. Interactions between the astroglial nuclear factor κB (NFκB)-mediated complement C3 release and neuronal C3aR receptors are crucial in changing the structure of dendrites and functioning of excitatory synapses [61]. Studies on APP/PS1 mice show that being the astroglia-specific NFκB target, complement protein C3 is required and sufficient to cause neuronal damage via binding to the C3aR on neurons with further intraneuronal calcium signaling [61]. Notably, several neurological disorders, including AD, have impaired synaptic function as a primary result of calcium dysregulation [62]. Thus, activation of the neuronal C3aR leads to an intraneuronal calcium increase, which, in turn, enhances synaptic excitation and impairs dendritic morphology, both of which contribute to neuronal dysfunction. Furthermore, the C3 produced by astrocytes also interacts with the C3aR on the microglia, exacerbating Aβ pathology. Since Aβ-induced dendritic and synaptic loss in the neurons is caused by NFκB/C3/C3aR signaling, it seems reasonable for C3aR antagonists to improve cognition in APP/PS1 mice [45,61]. These studies provide further evidence that Aβ-associated synaptic abnormalities and neuronal hyperexcitability depend on unique neuron–glia signaling pathways.

Moreover, the identification of terminal membrane attack complex (MAC), the final product of the activation of the complement pathways, near-fibrillar Aβ plaques, and synapses, highlighted the activation of not only the classical but all three complement pathways in AD [24]. In particular, APP knock-in (KI) mouse models of AD demonstrated elevated levels of MAC, C1q, and C3 in the brain synaptosomes [55]. The application of MAC-blocking antibodies, as well as the depletion of MAC component C6, confirmed the detrimental role of the MAC in synaptic loss [55], though the core mechanisms need further clarification.

Human studies have also confirmed the contribution of the MAC to synaptic pruning in AD. In the hippocampus and frontal cortex of AD patients, there was a significant reduction in both the mRNA and levels of complement defense 59 (CD59) protein, which prevents complement membrane attack complex (MAC) assembly [63]. However, the levels of complement component 9 (C9), the last component needed for the formation of the MAC, showed a significant increase. Furthermore, a strong association was discovered between the decline in CD59 and synaptophysin [63], suggesting that deficiencies in CD59 expression could lead to greater MAC generation and destruction of the synapses in AD.

Extensive reports highlight the important role complement proteins play in the inflammatory response in AD [64,65,66]. It has been hypothesized that complement dysfunction may cause neuroinflammation in an AD patient decades before clinical symptoms appear [6]. Evidence demonstrating that complement activation may speed up the development of AD leads to the idea that inhibiting complements could be a possible therapeutic strategy [54].

## 5. Is Complement Activation Beneficial or Detrimental in AD?

Determining whether complement activation is advantageous or harmful in AD is still highly debatable. In some experiments, complement deficiency has been shown to protect against Alzheimer’s disease, in some to have yet no effect, and to possibly worsen the condition in others [13,51,67]. Some authors claim that C1q deletion does not affect amyloid load, implying that C1q functions downstream of Aβ [24]. Furthermore, when the C1q complement component is inhibited, the microglia are unable to remove the glutamate-containing vesicular blebs produced by injured neurons and apoptotic cells [68]. Increased neurodegeneration was shown in the 3xTg mouse model deficient in C1q [69].

Other studies show that complement inhibition or deficiency causes accelerated amyloid pathology [51,59], emphasizing the role of C3 and CR3 in Aβ phagocytosis. Indeed, as fibrillar amyloid plaques are accessible to the complement components, activated complement components opsonize and facilitate their elimination [51]. Genetical studies have provided evidence that mutations in complement receptor 1 (C1R) may contribute to the progression of AD through modulation of Aβ accumulation [70]. A study carried out using 12- and 17-month-old amyloid precursor protein (APP)/C3−/− mice demonstrated the beneficial role of C3 in plaque clearance and neuronal health [51]. Damaged neurons are also not cleared and eliminated in C3-deficient animals. Likewise, in studies that used APP-transgenic mice and other models, C3 inhibition accelerated Aβ plaque deposition [51,71,72].

APPswe/PS1ΔE9 transgenic mice administered the C3aR1 antagonist SB290157 showed decreased microgliosis and Aβ pathology [64], in contrast to other findings that demonstrated decreased microgliosis and a decreased amyloid load according to treatment with a C3aR1 agonist [66]. Remarkably, it has been documented that when SB290157 is administered at a concentration of 10 μM, it functions as an agonist for C3aR1 instead of an antagonist [73,74], thus demonstrating the beneficial role of C3aR1 activation in AD. Furthermore, scientists showed that complement synthesis and C3aR1 activation via C3a control the expression of homeostatic genes during development, limiting alterations in the transcriptome of the microglia [66].

Low levels of CSF complement proteins have been positively correlated with faster cognitive deterioration and acceleration in the progression of AD according to recent studies [67]. A reduction in the levels of complement proteins has been partially linked to their capture in amyloid plaques. Additionally, decreased C1q levels were strongly correlated with a lower mental status and cognitive performance according to this same study. Similar findings have been reported by another cohort study using the Luminex assay, linking lower levels of CSF C3 to an accelerated decline in cognition [75].

Nevertheless, other animal models have shown that the complement plays a detrimental role in AD. Increasing evidence shows that along with diminishing the number of phagocytic microglia, the inhibition of the complement components C1q, C3, and CR3 also reduces the extent of synapse loss [76]. In an AD mouse model, C1q elimination (either through deletion or the use of C1q neutralizing antibodies) or reducing C3 enhanced the number of synapses and improved cognitive performance [77]. In other studies, C3 deficiency prevented the elimination of synapses from the damaged neurons compared to the control [78].

It is well established that the most vulnerable regions of the brain in terms of synapse loss are the hippocampus and frontal cortex. Interestingly, familial AD-mutant hAPP (“J20”) transgenic mice showed more pronounced C1q elevation and enhanced levels of the microglial lysosomal protein CD68 in these areas [12]. Moreover, the authors reported that C1q synaptic localization was increased even before plaques were formed [12]. Quantification of colocalized pre- and postsynaptic markers (synaptophysin and PSD95, synaptotagmin, and Homer) in these animals revealed a significant loss of synapses at 3–4 months old, an age that precedes plaque deposition. Furthermore, the microglia of wild animals that had been challenged with intracerebroventricular injection of soluble Aβ had much higher levels of CD68 immunoreactivity and more pronounced microglial pruning of the synapses and synaptic loss. However, C1q, C3, or C3 receptor knockout mice, as well as those receiving pharmacological treatment with an anti-C1qa-blocking antibody, had rescued synapse loss and did not show increased CD68 immunoreactivity in response to Aβ [12]. Additionally, the absence of C1q in the transgenic hAPP Tg2576 mouse model prevented the generation of the downstream products C3a and C5a of the classical complement pathway [79]. It also kept synaptophysin and MAP2 from being lost in the CA3 area of the hippocampus. These data provide evidence that synapse loss in AD is mostly caused by complement-dependent microglial clearance of the synapses.

On the other hand, the activation of the complement system has exacerbated tau pathology [1]. In a separate study, unbiased proteomic analysis of the postsynaptic densities (PSDs) of tau-P301S transgenic mice was used to uncover the tau-dependent synaptic changes before explicit neurodegeneration. Besides disturbances in other proteins and pathways, the authors observed the depletion of a set of GTPase-regulatory proteins involved in the rearrangement of the actin cytoskeleton, which eventually led to dendritic spine loss. In this study, complement C1q accumulation in the PSDs of the tau-P301S mice and AD patients has been correlated with the amount of phospho-tau. The accumulation of the complement C1q in the PSDs in these models resulted in augmented engulfment of the synapses by the microglia and a reduction in synapse density. Additionally, a C1q-blocking antibody reduced the microglial-induced synapse loss and restored the synapse density in tau-P301S mice as well as in cultured neurons, suggesting that the synaptic pruning initiated by tau pathology depends on C1q [78,80].

Additionally, C3 inhibition or knockout provides neuroprotection by inhibiting all the complement activation pathways [12,13,71]. In comparison to aged wild-type mice, aged C3 knockout mice showed increased long-term potentiation, implying increased synaptic activity and connectivity in the hippocampus and consequently an improvement in learning and memory. Accordingly, animals deficient in C3 did not show typical age-related hippocampus degeneration [71]. A similar effect was observed in the hippocampal CA3 region of APP/PS1 mice, which demonstrated the protective effects of C3 deficiency on synaptic and neuronal loss [13]. The consequence is clear: the complement may interfere with synaptic health in old age and AD [13].

Furthermore, microgliosis and astrogliosis in the hippocampus of animal AD models were alleviated, and the brain levels of pro-inflammatory cytokines were reduced by C3 loss in APP/PS1 mice. These aged APP/PS1; C3 KO mice exhibited an altered glial cell morphology and altered positions around plaques, demonstrating that C3 deficiency may influence the glial cell response to plaques [13]. These findings suggest that C3 may be a key factor in neuroinflammation that promotes synaptic dysfunction.

There is further debate around the effect of C3a and its C3a receptor on tau pathology. Recently, tau hyperphosphorylation induced by okadaic acid (OA), as well as the activity of glycogen synthase kinase 3β (GSK3β), was inhibited by an antagonist of the C3a receptor SB290157 [81]. The findings suggest that the C3a receptor has a unique role in controlling the phosphorylation of tau protein via the GSK3 signaling pathways [81]. In addition to a decrease in plaque-associated synapse loss in PS2APP animals, complement inhibition via deleting C3 also reduced neuron loss and brain atrophy in the Tau P301S mice. The authors stated that the enhanced levels of C3 in AD patients’ CSF correlate with tau accumulation and that diminishing C3 function may be helpful for other types of tauopathies [18].

The mechanisms by which the microglia and astrocytes ingest excitatory and inhibitory synapseshave been demonstrated in the PS19 tau line [81]. It is interesting to note that excitatory synapses have been found in the lysosomes of astrocytes, while microglial lysosomes contained only inhibitory synapses. Furthermore, the lack of C1q in the PS19 mouse models demonstrated ameliorated synaptic elimination and reduced pruning of the synapses by the microglia and astrocytes.

It should be highlighted, nonetheless, that not all the research has discovered a strong relationship between clusterin (CLU) and complement receptor 1 (CR1) and changes in CSF protein levels for Aβ and tau [82]. The reason for these contradictory results might be that many mice models used to replicate early-onset AD only contain single-gene alterations, as opposed to the prevalent multi-gene mutations observed in late-onset AD. Therefore, it is possible that the conclusions drawn from these mice models are limited in scope and cannot be applied to other cases.

On the other hand, the controverting conclusions coming from the published data can be explained by the varied experimental models (acute or chronic) utilized and different brain areas and different stages of disease studied. Moreover, in mouse models of amyloid or tau neuropathology, the developmental timing of C3aR1 ablation and the duration of the activation or inhibition of the C3aR1 pathway may be crucial factors. For instance, in a study by Lian et al. [61], short-term (1 h) treatment of the microglia with C3 or C3a enhanced phagocytosis, while longer treatment (24 h) reduced it [64]. Additionally, the beneficial effects of C3 on Aβ clearance have been shown in APP and hAPP mice older than 10 months with sustained Aβ plaque pathology [51]. However, the studies demonstrating the harmful effects of C1q, C3, and CR3 on synaptic homeostasis have investigated the pre-plaque stages of the disease in J20 [12] and APP animals [83].

## 6. Complement Components at Different Stages of AD

**Figure 2:**
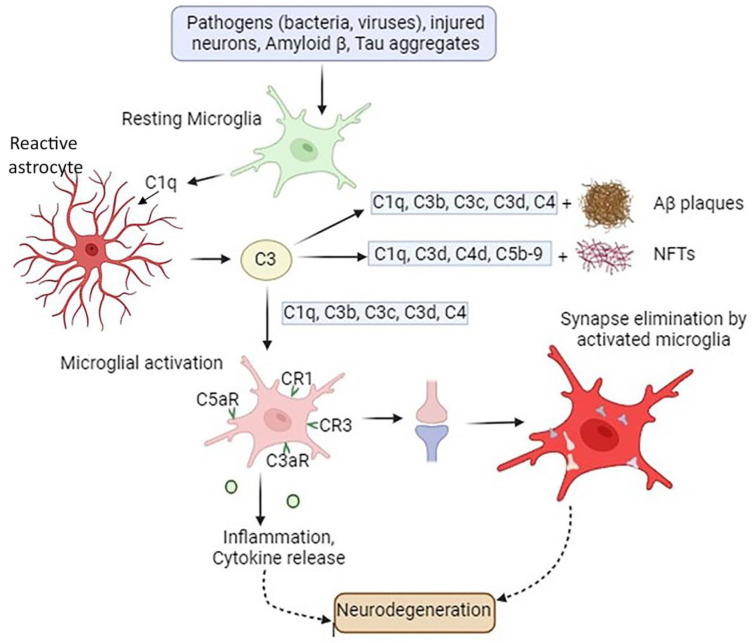
Involvement of complement proteins in AD pathogenesis. In the presence of pathogens such as bacteria or viruses in the CNS, signals released by injured tissue, apoptotic neurons, Aβ protein, and tau aggregates cause the microglia to produce C1q, along with other molecules. This is followed by the release from astrocytes of C3 complement protein, which co-localizes with NFTs, weakened synapses, and amyloid plaques. Complement components C1q, C3b, C3c, and C3d accumulate in amyloid plaques and dystrophic neurites, while complement proteins C1q, C3d, C4d, and C5b-9 are deposited onto neurofibrillary tangles (NFTs). Both amyloid plaques and NFTs initiate microglial activation. Additionally, microglia possess complement receptors CR1, CR3, C3aR, and C5aR1, which allow these cells to bind various complement opsonins, such as C1q, C3b, iC3b, C3d, C4b, C3a, and C5a. These interactions result in microglial activation, followed by synaptic elimination, chronic inflammation, and exacerbation of neurodegeneration.

There are still many significant unanswered concerns regarding the molecular mechanisms that underlie the complement cascade’s regulation and impact on neuronal function and dysfunction in AD brains. It has been revealed that minor memory loss at the initial stage of AD is directly related to synapse loss and neuron dysfunction and is caused by the classical complement cascade [12]. Studies show that, initially, the Aβ peptides attach to a collagen-like domain (CLF) within C1q (Figure 2). Because the binding of Aβ 1-42 to C1q is more effective than that of Aβ 1-40, this pathway has more impact on Aβ 1-42, resulting in its assembly into aggregates and fibrils. This interaction leads to the activation of the classical complement pathway and results in synapse loss. Indeed, the C1q, as well as TNF-α and interleukin-1α (IL-1α), released from activated microglia promote astrocytes to change to their reactive pattern, namely becoming A1 astrocytes, which are the main producers of complement protein C3 [84]. The complement C3 released from A1-reactive astrocytes accumulates in amyloid plaques, NFTs, and weakened synapses [45].

The question is whether the synaptic pruning mechanism is helpful initially but subsequently becomes dysregulated in the long term and chronically, impairing the neurons it aims to conserve. The steps in the activation of the complement cascade on different stages of AD have been reported in many studies [4,5]. It has been shown that at the early stages of the disease, C1q expression is increased by tissue injury, amyloid oligomers, apoptotic neurons, and neuronal blebs. The binding of C1q to these structures initiates phagocytosis on the part of the microglia. In the absence of other pathogens or damage-associated molecular patterns, this causes anti-inflammatory activity.

The accumulation of fibrillary Aβ and local damage, however, leads to further C1r, C1s, C4, and C3 synthesis and chronic activation of the complement cascade. Newly generated C3b/iC3b is covalently bound to fibrillary Aβ and may lead to phagocytosis via CR3. Nevertheless, at the more advanced stages, in addition to 2b, 3b, and 4b being bound to fibrillary Aβ, C5a and C5b are also generated. C5a induces chemotaxis in the microglia, providing their recruitment to the amyloid plaques. C5a diffuses from the plaques, binds to the anaphylatoxin receptor C5aR1 on the microglia, and induces a chronic inflammatory state by acting on MAPKs and activating the production of pro-inflammatory mediators [4]. According to Carvalho et al., [85] the primary way that C5a-C5aR1 signaling works in AD is via stimulating pathways that activate the microglia, leading to disease progression. As was mentioned, the fibrillary Aβ also binds to the microglial TLR receptors. Interestingly, C5a–C5aR1 signaling was found to synergize with TLR2 and TLR4 [86]. Acting in concert, they enhance pro-inflammatory cytokine responses and reactive oxygen species production, leading to a neurotoxic environment. Thus, while the binding of C5a and C3a recruits phagocytic cells to the plaques, the intracellular signaling of C5a and C3a on the microglia induces a chronic inflammatory state [87]. AD patients had higher serum levels of C5a, TNF-α, IL-1β, IL-6, and CRP than non-AD subjects did [5]. Furthermore, more severe AD patients demonstrated higher levels of C5a compared to patients with mild and moderate AD. These levels had a positive correlation with plasma pro-inflammatory factor levels and a negative correlation with cognitive function [5]. Moreover, C5a initiates apoptosis in the neurons [85], and C5a/C5aR1 signaling increases vascular permeability by causing endothelial cell apoptosis, further exacerbating brain pathology [88].

Consequently, in large quantities, fibrillary Aβ plaques cannot be properly removed from the brain tissue, and a chronic inflammatory environment develops, contributing to more fibrillary Aβ production, greater neuronal damage, and death.

## 7. Complement-Modulating Approaches in AD Treatment

Increasing evidence shows that selective genetic or antibody-based complement inhibitory approaches that protect the synapses and memory loss can be beneficial in slowing down the progression of AD [54,55]. For instance, the overexpression of CD55, an inhibitor of the complement pathways produced in the neurons upon inflammation, resulted in the alleviation of synaptic loss in the dentate gyrus of the hippocampus [89]. This also led to fewer synaptic dissociations in the CA1 area of the rodent hippocampus and less remote memory loss [89].

Blocking the signaling of the complement receptors C3aR and C5a-C5aR1 reduces synaptic pruning and the amyloid burden, slows the appearance of plaque-associated dystrophic neurites, and avoids microglial polarization toward a more detrimental disease-associated inflammatory phenotype, allowing for inhibition of inflammation in AD mouse models. Even though all the upstream complement components involved in synaptic pruning were still there, cognition was better and the AD pathology was less severe when the C5a-C5aR1 interaction was blocked using drugs or deleted genetically [86]. The Aβ-induced inflammatory response initiated by C5a was inhibited by C5a-targeting vaccines, as well as by PMX205, a C5aR antagonist, in two mouse models of AD (Tg2576 and 3xTg) [90]. The animals showed improved contextual memory and reduced cerebral amyloid plaques. Since JAK/STAT3 signaling is involved in neuroinflammatory responses to Aβ and C5a, inhibiting this route using an antagonist, AG490, prevented the generation of pro-inflammatory cytokines as well [91]. In other studies, PMX205 treatment reduced microglial and astroglia activation, as well as diminishing fibrillar amyloid accumulation and cognitive loss [79]. In the 3xTg models, which are characterized by tangle accumulation, PMX205 treatment caused hyperphosphorylated tau to drop by 70%. Moreover, the cytotoxic and inflammatory effects of Aβ42 on microglial BV-2 cells that were initiated by C5a were also blocked by the C5aR antagonist PMX205 [91].

Furthermore, neuronal damage was prevented by the C5aR1 antagonist PMX53, a near analog of PMX205. The primary neurons obtained from C5aR1 null mice showed reduced Aβ42-induced damage after being exposed to the maximum C5a dose examined. The authors speculated that the C5aR1 antagonist’s positive benefits in AD mice models may have been because it protects the neurons from C5a’s harmful effects [92].

The beneficial effects of PMX205 and PMX53 on the loss of neurons have been also demonstrated in other neurodegenerative diseases and culture models [4,92]. In hSOD1G93A mice, a model of amyotrophic lateral sclerosis (ALS), Lee et al. [93] used PMX205 as a C5aR antagonist. According to their study, oral administration of PMX205 increased the animals’ grip strength, retarded the progression of the disease, and increased their chances of survival by penetrating the brain at a pharmacologically effective concentration [93]. In Tg2576 animals, the antagonist prevented the loss of pre-synaptic markers and decreased dystrophic neurite and Aβ levels [94]. Additionally, it partially restored microglial homeostatic genes and alleviated the memory loss associated with AD [94].

Another experiment was conducted on a transgenic AD model that contained a third “Arctic” mutation in the human APP transgene with a high production of fibrillar Aβ plaques. Deletion of the C5aR1 gene rescued the loss of the neuronal complexity in the CA1 region of the hippocampus and behavioral deficits observed in C5aR1-sufficient Arctic mice at 10 months of age, without any change in plaque accumulation [92].

Carvalho et al. [85] demonstrated that in AD models, C5a-C5aR1 signaling mostly affects disease progression by accelerating the microglial activation pathways [85]. Microglial activation and astrogliosis were reduced in C5a-deficient mice [94]. Meanwhile, the interaction of C5a with C5aR2 demonstrates a neuroprotective function, and the binding of C5a to C5aR1 results in disease progression. In Arctic mice with C5a overexpression, the authors showed that specific pharmacological inhibition of C5aR1 could be a potential treatment strategy for AD [85]. In a different study, which used the AD amyloidosis mouse model (5xFAD model), it was demonstrated that EP67, a modified C5a receptor agonist, increased the clearance of both fibrillar and non-fibrillar Aβ by the microglia, therefore mitigating synaptic degeneration and ameliorating cognitive impairment [95].

The deletion of C5AR1 and TYROBP in other mice with modified inflammatory processes showed a restoration of the gene expression circuits and cognitive decline. However, there was no decrease in amyloid plaque deposition [96]. These findings are similar to reports of cognitively normal people, whose brains demonstrated significant plaque pathology at autopsy [97], implying that it is the induced response to the plaques rather than the plaques themselves that is harmful in AD. Moreover, C5aR1 deletion restricts the polarization of the microglia to more harmful disease-associated inflammatory cells in animal models of AD, allowing for continued phagocytosis and degradation with fewer inflammatory side effects [86]. Overall, the data from mice and people suggest that blocking C5aR1 is protective and may reduce inflammation and improve homeostasis in AD.

Finally, recent research has found that C8γ, one of the three subunits of complement protein C8 (α, β, γ) which makes up the MAC, could be a promising therapeutic target for AD and other neurological conditions [98]. The authors identified the inhibitory effects of C8γ on glial hyperactivation, neuroinflammation, and cognitive loss. The pathophysiology of Alzheimer’s disease is correlated with impaired autophagy in the glial cells, and therefore understanding the molecular mechanisms of such autophagy dysfunction is a prerequisite for the development of new therapeutic strategies [99].

Nevertheless, additional investigation into the mechanisms governing complement-mediated synaptic loss is essential to identify potential biomarker candidates that could aid in the early diagnosis of complement dysregulation, slow the disease’s progression, and potentially enhance the cognitive and memory performance of AD patients in the future.

## 8. Conclusions, Challenges in Targeting the Complement, and Future Perspectives

Depending on the degree of activation and the initial targets, the interactions between complement components and activated microglia can be neurotoxic, causing synaptic loss, neuroinflammation, and ultimately progressive neurodegeneration. By mitigating beta-amyloid-mediated complement system activation the anti-inflammatory treatments show positive effects against synaptic loss in AD. Additionally, the insights from synapse pruning in normal brain development offer promising drug targets to preserve cognition in AD before plaque formation. However, the complement system is intricate, with multiple components and regulatory mechanisms, which understanding and precisely manipulating are challenging. Therefore, targeting the molecular mechanisms of complement-driven, microglia-mediated synaptic loss in Alzheimer’s disease requires further investigations.
